# There is a need for more precise models to assess the determinants of health crises like COVID-19

**DOI:** 10.3389/fpubh.2023.1179261

**Published:** 2023-06-15

**Authors:** Alessandro Rovetta

**Affiliations:** R&C Research, Research and Disclosure, Brescia, Italy

**Keywords:** COVID-19, epidemiology - analytic (risk factors), public health, public health policies, epidemic determinants, confounding (epidemiology)

## Abstract

The COVID-19 pandemic has had a significant impact on global mortality. While the causal relationship between SARS-CoV-2 and the anomalous increase in deaths is established, more precise and complex models are needed to determine the exact weight of epidemiological factors involved. Indeed, COVID-19 behavior is influenced by a wide range of variables, including demographic characteristics, population habits and behavior, healthcare performance, and environmental and seasonal risk factors. The bidirectional causality between impacted and impacting aspects, as well as confounding variables, complicates efforts to draw clear, generalizable conclusions regarding the effectiveness and cost-benefit ratio of non-pharmaceutical health countermeasures. Thus, it is imperative that the scientific community and health authorities worldwide develop comprehensive models not only for the current pandemic but also for future health crises. These models should be implemented locally to account for micro-differences in epidemiological characteristics that may have relevant effects. It is important to note that the lack of a universal model does not imply that local decisions have been unjustified, and the request to decrease scientific uncertainty does not mean denying the evidence of the effectiveness of the countermeasures adopted. Therefore, this paper must not be exploited to denigrate either the scientific community or the health authorities.

## Introduction

The dramatic impact of COVID-19 on global mortality is a scientific fact (1–3). Indeed, the size and significance of the effect are so large and in agreement with the vast literature on the subject that bias analysis is not needed to ascertain the mere existence of this causal relationship (4). However, more precise and complex models are required to attribute the exact weight to all the epidemiological factors involved. Indeed, although it is true that the ability of a pathogen to compromise public health—in all its aspects—is part of its inherent hazard (e.g., overloading of health facilities due to high contagiousness and virulence), it is also true that such a threat is determined by the variables that it affects (e.g., the performance of health facilities). Moreover, COVID-19 behavior appears to be influenced by a wide range of risk factors and determinants, the assessment of which is undermined by known confounders and bidirectional relationships (Table 1). Specifically, in such a mathematically and scientifically complex system, constructing a global statistical cost function and ascertain primary causes of phenomena can be demanding. Since these elements are essential to draw up a prioritization scheme of interventions (i.e., which variables to tackle or influence first to obtain the best benefit) as well as a methodology of intervention (i.e., how to tackle or influence a specific variable to obtain the best benefit), the whole public health decision-making process is potentially compromised and/or severely slowed down. To the best of the authors' knowledge, no current model satisfactorily incorporates all these variables. This also makes scientific conclusions, and therefore public health actions, varyingly exposed to authors' interpretations and biases (5).

**Table 1:** COVID-19-related epidemiological variables.

**Impacted/ing aspects**	**Determinants and risk factors**	**Confounders**
- Availability of care beds - Availability of health personnel - Availability of medical equipment for treatments - Availability of drugs - Availability of protective equipment - Performance of healthcare personnel - Performance of healthcare systems	- Age (weaker immune system) - Gender - Pre-existing medical conditions - Population habits and behavior - Poor healthcare capacity and/or quality - Health infodemic - Pollution and other environmental factors - Seasonal risk factors	- Historical differences in determinants and risk factors - Historical differences in healthcare capacity and/or quality - Undesired NPC impact on healthcare system - Undesired NPC impact on people health - Undesired NPC impact on contagion - Asymptomatic cases - Testing capacity and/or quality

### Impacted and impacting aspects

A very contagious and aggressive virus like SARS-CoV-2 can impair the health system causing facilities overload (6), shortage of healthcare personnel (7, 8), shortage of medical-related supplies (6, 9), physical and mental exhaustion of healthcare workers (10), and other human errors in managing the emergency (10, 11) (impacted aspects). At the same time, poor healthcare can obviously increase COVID-19 (and other diseases) severity, fatality, and mortality. For this reason, causality between impacted and impacting aspects is bidirectional and subject to confounding.

### Determinants and risk factors

The scientific literature on COVID-19 reports various risk factors related to the individual's health status, including age, gender, and a long list of specific pre-existing conditions (12–15), population habits, movements and adherence to anti-pandemic regulations (15, 16), insufficient or delayed healthcare, information overabundance and success of misleading and/or incorrect news (even among healthcare workers) (17), air pollution (18), environmental and meteorological factors including temperature, relative humidity, sunlight, and wind (19–22), and seasonal risks such as the arrival of cold weather (19, 20).

### Confounders

Historical differences in risk factors and health service adequacy may create apparent differences in virus fatality and severity as intrinsic epidemiological characteristics. Furthermore, lockdowns and social distancing—considered by the majority of the scientific community as an essential tool for the containment of the infection (23–25)—have caused heterogeneous detrimental effects, varying in effect size and prevalence, both at the socio-psychological level (26–28) (which can have repercussions on physical health), in healthcare services, and even contagion dynamics (29). Alongside this, asymptomatic cases, insufficient testing capacity and quality can further bias the estimation of deaths possibly associated with COVID-19 (30–33).

### Recommendations

In light of this evidence, I ask that the scientific community and health authorities worldwide begin to develop comprehensive models not only for the current pandemic emergency but also for future health crises. Considering a typical epidemiological study design, this means conducting a thorough literature search on all known or suspected variables that may potentially interact with the pathogen of interest. This also means developing multivariable models with parameters based on local empirical characteristics (from the availability of drugs to suspected evolutionary mutations) to determine the epidemiological role and weight of each variable by fitting the observed data. One possible approach to achieving this goal is to use mixed models with reciprocal effects, training established algorithms (e.g., hierarchical regression and extended SEIR) enhanced with artificial intelligence (e.g., machine learning and neural networks) on both historical and current data (34–38). Naturally, bias analysis and expertise play a crucial role in accurate implementation. This also calls for further research on mathematical-epidemiological modeling of human aspects in various contexts, including general (e.g., people behavior), professional (e.g., hospital assistance dynamics), psychological (e.g., psychological reactions), and infodemiological (e.g., the effects of infodemics on concrete actions). Although the inclusion of all relevant variables may be an unattainable objective, successful modeling of some additional single or even groups of factors would allow for a better estimation of the impact of the remaining (un-modeled). The sensitivity analysis should be utilized to assess the reliability and predictive power of the models, as well as to examine the intercorrelations among inputs and outputs (39). Besides, the mission should not solely be to ensure the short-term survival of as many people as possible, but rather to seek a solution that ensures a sustainable lifestyle (i.e., both socially and psychologically viable). For example, by establishing varying degrees of lockdown severity and quality of life, the aim should be to scientifically establish the minimum severity level at which the mere epidemiological risk and quality of life are deemed acceptable. Such a point is vital for the success of long-term policies since people's adherence is strongly affected by pandemic fatigue and similar phenomena (40–42). Thus, decisions should be made based on the related cost functions. Indeed, at present, it is challenging to draw clear, unequivocal, and generalizable conclusions not so much on the effectiveness as on the cost-benefit ratio of non-pharmaceutical health countermeasures. Likewise, comparisons between countries' policies are also often arbitrary. In this scope, such models should be implemented locally to account for micro-differences in epidemiological characteristics that may have relevant effects (e.g., evolutionary mutations and/or particularly polluted areas that increase the pathogen virulence in a certain region). By doing so, it would be possible to ensure and protect public health in a timely manner based on the best available scientific evidence, minimizing the epidemiological impact and uncertainty in decision-making thanks to more targeted and specific investigations and interventions. This could also lead to greater trust in institutions (which could plausibly translate into greater adherence to required health regulations) and a decrease in fallacious and misleading debates on counterfactual scenarios (e.g., what would have happened if...). Undoubtedly, such a strategy would necessitate increased investment in local resources, such as surveillance systems and appropriately trained personnel. However, comparing provincial, regional, and national models could yield valuable insights into their differences and similarities, allowing for a better understanding of which factors require local analysis versus those that can be effectively modeled at larger scales. Whether this paper is too ambitious or not, the above considerations highlight that it is paramount to establish a theoretical goal to strive for and to call for moderation among those scientists who express too much certainty on inherently dubious topics, risking fostering distrust toward institutions and science (43, 44). Finally, I conclude by saying that this perspective must not be exploited to denigrate either the scientific community or the health authorities since (i) the lack of a universal model does not in any way imply that local decisions have been unjustified, and (ii) the request to decrease scientific uncertainty does not mean denying the evidence on the effectiveness of the countermeasures adopted but only expecting greater precision in ascertaining the entity of costs and benefits for future implementations. In this regard, the author of this paper expresses their solidarity with the victims of the COVID-19 pandemic phenomenon and with those who made great responsibility decisions disposing only of limited and uncertain data during a period of extreme social tension.

## Data availability statement

The original contributions presented in the study are included in the article/supplementary material, further inquiries can be directed to the corresponding author.

## Author contributions

The author confirms being the sole contributor of this work and has approved it for publication.
